# Association between sleep duration and antibody acquisition after mRNA vaccination against SARS-CoV-2

**DOI:** 10.3389/fimmu.2023.1242302

**Published:** 2023-12-11

**Authors:** Muneto Izuhara, Kentaro Matsui, Takuya Yoshiike, Aoi Kawamura, Tomohiro Utsumi, Kentaro Nagao, Ayumi Tsuru, Rei Otsuki, Shingo Kitamura, Kenichi Kuriyama

**Affiliations:** ^1^ Department of Sleep-Wake Disorders, National Institute of Mental Health, National Center of Neurology and Psychiatry, Kodaira, Tokyo, Japan; ^2^ Department of Clinical Laboratory, National Center Hospital, National Center of Neurology and Psychiatry, Kodaira, Tokyo, Japan; ^3^ Department of Psychiatry, The Jikei University School of Medicine, Minato-ku, Tokyo, Japan; ^4^ Department of Psychiatry, National Center Hospital, National Center of Neurology and Psychiatry, Kodaira, Tokyo, Japan; ^5^ Department of Psychiatry, Nihon University School of Medicine, Itabashi-ku, Tokyo, Japan

**Keywords:** mRNA SARS-CoV-2 vaccine, sleep and immunity, longer objective sleep duration, antibody titer, BNT-162b2, mRNA-1273

## Abstract

**Introduction:**

Sleep enhances the antibody response to vaccination, but the relationship between sleep and mRNA vaccination against severe acute respiratory syndrome coronavirus 2 (SARS-CoV-2) is not fully understood.

**Methods:**

In this prospective observational study, we investigated the influence of sleep habits on immune acquisition induced by mRNA vaccines against SARS-CoV-2 in 48 healthy adults (BNT-162b2, n=34; mRNA-1273, n=14; female, n=30, 62.5%; male, n=18, 37.5%; median age, 39.5 years; interquartile range, 33.0–44.0 years) from June 2021 to January 2022. The study measured sleep duration using actigraphy and sleep diaries, which covered the periods of the initial and booster vaccinations.

**Results:**

Multivariable linear regression analysis showed that actigraphy-measured objective sleep duration 3 and 7 days after the booster vaccination was independently and significantly correlated with higher antibody titers (B=0.003; 95% confidence interval, 0.000–0.005; Beta=0.337; p=0.02), even after controlling for covariates, including age, sex, the type of vaccine, and reactogenicity to the vaccination. Associations between acquired antibody titer and average objective sleep duration before vaccination, and any period of subjective sleep duration measured by sleep diary were negligible.

**Discussion:**

Longer objective, but not subjective, sleep duration after booster vaccination enhances antibody response. Hence, encouraging citizens to sleep longer after mRNA vaccination, especially after a booster dose, may increase protection against SARS-CoV-2.

**Study registration:**

This study is registered at the University Hospital Medical Information Network Center (UMIN: https://www.umin.ac.jp) on July 30, 2021, #UMIN000045009.

## 1 Introduction

Sleep and immunity are closely interrelated (1); the immune system is implicated in sleep regulation, while sleep affects immune functions against pathogens and vital homeostasis. Sleep restriction interventional and observational studies emphasize the need of longer sleep duration for adequate antibody acquisition (2–5). However, these studies were based on inactivated vaccines. The interaction between sleep and immune response to mRNA vaccines against severe acute respiratory syndrome coronavirus 2 (SARS-CoV-2) may differ; the injected mRNA molecule translates into the target protein for a longer time than that in inactivated vaccines (6). Although research needs have been stressed (7, 8), the relationship between sleep and mRNA vaccination against SARS-CoV-2 has not been thoroughly examined.

Objective sleep duration, measured using tools such as polysomnography and actigraphy (9), sometimes contrasts with subjective sleep duration, which is based on individual self-reports through sleep diaries (10). A recent meta-analysis showed that objectively measured short sleep duration using actigraphy was associated with a robust reduction in antibody response; however, this association was not observed when sleep duration was self-reported using sleep diaries (11). Therefore, despite a previous study indicating no association between subjective sleep duration and antibody titers (12), there’s a clear need for more studies utilizing objective sleep measurements.

To address this, we conducted a prospective observational cohort study using both actigraphy and sleep diaries to assess sleep duration. This study included both the initial and booster vaccinations to elucidate the influence of sleep habits on immune acquisition induced by mRNA vaccines against SARS-CoV-2.

## 2 Materials and methods

This prospective observational study was conducted between June 30, 2021, and January 26, 2022, at the National Center of Neurology and Psychiatry Hospital, a territorial psychiatry and neurology hospital in Tokyo, Japan. The study followed the Strengthening the Reporting of Observational Studies in Epidemiology (STROBE) reporting guidelines.

### 2.1 Participants

We recruited healthy participants aged 20–60 years by advertising. The exclusion criteria were regular medication use, history of SARS-CoV-2 infection, history of COVID-19 vaccination, and history of any vaccination within 1 month before the observation started. Participants received a 10,000-yen gift certificate after completion of the study for reimbursement and subsistence costs. The Ethics Committee of the National Center of Neurology and Psychiatry approved this study (approval no. A2021-040). Written informed consent was obtained from all participants. The study protocol was registered with the University Hospital Medical Information Network Center (https://www.umin.ac.jp) on July 30, 2021 (#UMIN000045009), prior to the start of the observation. Of the 50 participants recruited, two were excluded (one refused to take the vaccine, and the other was infected by SARS-CoV-2 with an elevated anti-N protein antibody titer). The remaining 48 participants were included in the analysis (**Supplementary Figure 1**), of whom 34 and 14 were vaccinated with BNT-162b2 and mRNA-1273, respectively. The vaccination interval was 21–35 days for BNT-162b2 and 28 days for mRNA-1273.

### 2.2 Antibody titer

The main outcome, antibody titers against the receptor binding domain of the SARS-CoV-2 spike (S) protein, was measured using an anti-SARS-CoV-2 S enzyme immunoassay (Elecsys Anti-SARS-CoV-2 S; Roche Diagnostics, Indianapolis, IN, USA). In addition to spike protein, titers of antibodies against the nucleocapsid (N) protein were also evaluated using an anti-SARS-CoV-2 enzyme immunoassay (Elecsys Anti-SARS-CoV-2; Roche Diagnostics) to exclude those previously infected participants with SARS-CoV-2.

Blood samples were collected from participants into serum-gel tubes according to standardized operating procedures. Samples were centrifuged at 2000 g for 10 min. Samples were stored in the laboratory refrigerator at −80°C until laboratory testing was conducted. Blood samples were collected in the laboratory of the National Center of Neurology and Psychiatry. Titer measurements of blood samples were performed at local clinical laboratories in Hino, Tokyo, Japan (SRL Laboratory Inc., Hino, Tokyo, Japan).

Serum samples were tested using the automated serological immunoassays, Elecsys ^®^ Anti-SARS-CoV-2 S (Elecsys^®^ anti-S, Cat # 0928926750) and the Elecsys^®^ Anti-SARS-CoV-2 (Elecsys^®^ anti-N, Cat # 09203095501), by detecting antibodies to the receptor-binding domain (RBD) of S protein and antibodies to the N protein of SARS-CoV-2 (Roche Diagnostics) (13).

Serology samples were tested for total (IgG, A, and M) anti-SARS-CoV-2 spike (S) antibodies and anti-nucleocapsid (N) antibodies using Roche Elecsys-S/Elecsys Anti-SARS-CoV-2 assays, respectively. The results by Elecsys^®^ anti-S were quantified in units of U/mL with the cut-off point of 0.80 U/mL to differentiate samples as reactive (≥ 0.80 U/mL) and non-reactive (< 0.80 U/mL) (14). The Elecsys^®^ anti-N is a semi-quantitative assay, and the results were interpreted as follows: cutoff index (COI) <1.0 was non-reactive, and ≥1.0 was reactive (15). We did not measure antibodies to the SARS-CoV-2 spike protein (anti-S antibodies) at study initiation because the titers were expected to be below the sensitivity levels of the anti-S antibody test. The level of anti-S antibodies only increases after vaccination or infection (16). At the study baseline, none of the participants were vaccinated against COVID-19. Furthermore, participants showing signs of SARS-CoV-2 infection were excluded based on the elevation of antibodies against the N protein (anti-N antibodies) (16).

Participants were vaccinated twice with either BNT-162b2 (Pfizer-BioNTech; BioNTech, Mainz, Germany) or mRNA-1273 (Moderna, Cambridge, MA, USA). The vaccination interval was 21 days for BNT-162b2 and 28 days for mRNA-1273. Cross-inoculation was not allowed. We collected blood samples between 1 and 3 weeks following booster vaccination, considering the anticipated peak in the antibody titer (17, 18).

### 2.3 Sleep measurements

Participants recorded their objective sleep using activity-based sleep recordings (actigraphy). This data collection started from the date of consent and continued until 2 weeks after the booster vaccination, thereby covering a period of 3 weeks prior to and 2 weeks following the booster vaccination. MTN-210 (Kissei Comtec, Nagano, Japan) was used for activity-based sleep recordings (19). The participants attached the MTN-210 to the front of the trunk by clipping it to the edges of the trousers/pants and wearing it for 24 hours daily. The MTN-210 is 27 mm in diameter, 9.1 mm thick, and weighs 9 g including the battery and records the amount of activity using an internal three-axis accelerometer (electrostatic capacitance sensor). Every 0.125 seconds, the number of times the acceleration exceeds a reference value is summed and recorded as an activity value over 2-minute bins. The activity intensity is calculated from the activity value as a value from 0 to 63 (64 steps). An activity intensity of 0 means that the subject did not perform any movement, and higher values indicate a higher level of activity. A laboratory validation study showed 84.7–85.4% agreement with polysomnography data when the MTN-210 was attached to the front of the trunk (19). Data were extracted from the MTN-210 devices through a near-field communication interface (PaSoRi, RC-S380; Sony Corporation, Tokyo, Japan) using SleepSign Act software (Kissei Comtec). For sleep/wake detection from the MTN-210 data, the default settings in SleepSign Act were used, in which sleep detection followed the previously reported algorithm (19). It is considered insufficient if data loss exceeded 20% (20). Sleep diaries were used to measure subjective sleep patterns (10). The participants were required to estimate the sleep duration every morning and record their findings in the sleep diary. Participants with missing actigraphy data were retained in the analyses using mean substitution.

### 2.4 Other covariates

Data on previously detected factors influencing vaccine responses were also collected, including age, sex, type of mRNA vaccine (17, 18, 21), and systemic reactogenicity (22–24). We also recorded the time of day of vaccination, as previous studies with inactivated vaccines have demonstrated an interaction between immune response and time of vaccination (25).

### 2.5 Reactogenicity

Studies have previously reported a correlation between antibody titers induced by mRNA vaccines against COVID-19 and systemic reactogenicity owing to booster vaccination (22–24). We asked participants to evaluate the worst systemic symptoms (highest temperature, dizziness, fatigue, headache, chills, nausea, diarrhea, muscle pain, and joint pain) for 8 days following booster vaccination, according to the US Food and Drug Administration grades of systemic reactogenicity, ranging from 0 to 3 (0, no symptoms; 1, no interference with activity; 2, some interference with activity but not requiring medical intervention; 3, preventing daily activity and requiring medical intervention) (26). Body temperature was rated as follows: grade 1 (mild), 38.0°C–38.4°C; grade 2 (moderate), 38.5°C–38.9°C; grade 3 (severe); ≥39.0°C.

### 2.6 Sample size calculation

We assumed an effect size of 1.3 to estimate the sample size based on a previous study that compared vaccine titers induced by the hepatitis B vaccine between participants who slept ≤6 hours and those who slept ≥7 hours (4). The study needed 14 participants to achieve 95% power with a two-sided alpha level of 0.05. The proportions of people who slept ≤6 hours and those who slept ≥7 hours were 37% and 25%, respectively, according to a national nutrition survey conducted by the Japanese Ministry of Health, Labor and Welfare (27). A 6.7% dropout rate was estimated; therefore, 60 participants were planned for enrollment.

### 2.7 Data analysis

We used log transformation of antibody titers for better normal distribution. Fisher’s z-transformation was employed to mitigate the differences between the two mRNA vaccines (28, 29). Linear fitting and correlation coefficient (R^2^) calculation were conducted to examine the relationship between actigraphy measured objective sleep duration 3 days following booster vaccination and log-transformed antibody titers.

For the main analysis, all variables were initially assessed using univariable linear regression. We then performed hierarchical linear regression analyses to examine the relationships between post-vaccination antibody levels and sleep measures, which showed significant correlations with the antibody titer in the univariable linear regression, controlling for previously reported covariates associated with immune response (17, 18, 21–24). For model 1, we performed multivariable linear regression analysis with age and sex as covariates. For model 2, we performed another multivariable linear regression analysis including age, sex, vaccine type, and systemic reactogenicity as covariates. In our regression analyses, ‘B’ refers to the unstandardized coefficients, which indicate the unit change in the dependent variable for a one unit change in the predictor variable. ‘Beta’ represents the standardized coefficients, which are a measure of how many standard deviations the dependent variable will change per standard deviation increase in the predictor variable. To assess the relationship between antibody titer and average sleep duration, we divided the sleep data into four time bins for both actigraphy and sleep diary. Following the protocol of a previous study (4), we examined average sleep duration for 3 days before and after each vaccination. In addition, given that mRNA vaccines can produce antigens for ≥1 week (6), we examined the average sleep duration for 7 days before and after each vaccination, as well as for the entire observation period.

All statistical analyses were performed using SPSS version 23 (IBM Corp., Armonk, NY, USA). All statistical tests were two-sided, with p<0.05 indicating statistical significance.

## 3 Results

**Figure 1:**
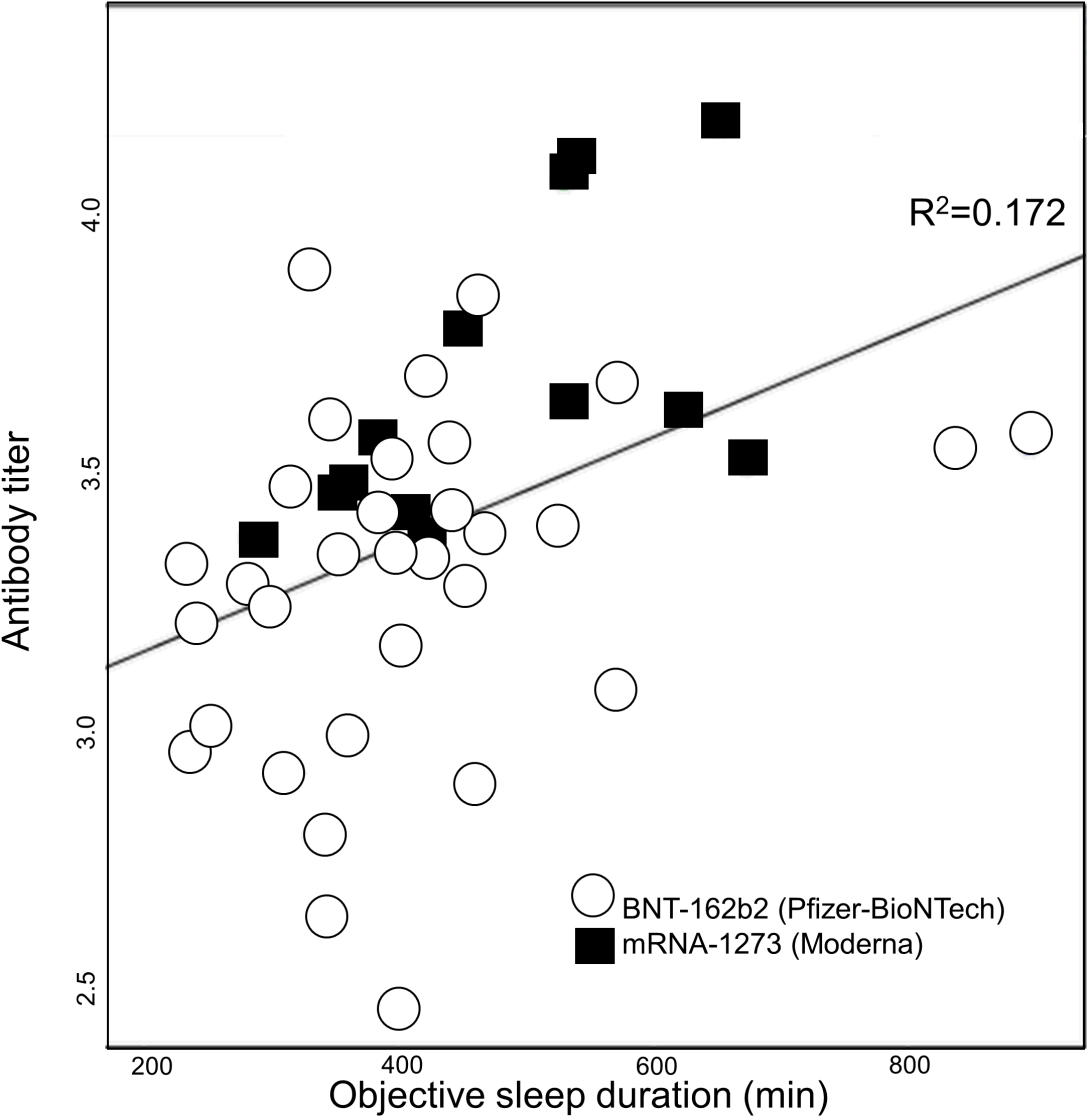
Average sleep duration 3 days following booster vaccination and log-transformed antibody titers. The line represents the linear regression line fitted to the data.

The background characteristics of the participants are summarized in **Table 1**. Participants were 62.5% female (n=30), with median age of 39.5 (interquartile range, 33.0–44.0) years. All the participants used sleep diaries to record their sleep duration during the observational period. The daily percentages of participants without actigraphy data over a two-week period before and after the initial and booster vaccination dates are shown in **Supplementary Table 1**. Over 80% of the actigraphy data were collected from 1 day before the first vaccination to 11 days after the booster vaccination. Conversely, less than 80% of the actigraphy data was collected during two specific periods: 2 or more days before the first vaccination and 12 or more days after the booster. The scatter plot of actigraphy data of average sleep duration and antibody titers is shown in **Figure 1**. In addition, the separate scatter plots for BNT-162b2 and mRNA-1273 are shown in **Supplementary Figure 2** and **Supplementary Figure 3**, respectively.

**Table 1:** Participant characteristics.

	Type of vaccine		Total (n=48)
	BNT-162b2 (n=34)	mRNA-1273 (n=14)	
Sex, females	20 (58.8)	10 (71.4)	30 (62.5)
Age (years)	40.5 (32.8–44.0)	35.5 (32.3–45.5)	39.5 (33.0–44.0)
Systemic reactogenicity^a^	5.0 (2.0–13.3)	14.5 (9.5–27.0)	7.0 (3.0–15.8)
Fever	0.0 (0.0–0.0)	2.0 (0.8–3.3)	0.0 (0.0–1.0)
Dizziness	0.0 (0.0–0.0)	0.0 (0.0–2.0)	0.0 (0.0–0.0)
Fatigue	1.0 (0.0–2.0)	4.0 (1.8–6.5)	1.0 (0.0–4.0)
Headache	1.0 (0.0–2.0)	4.0 (1.5–6.3)	1.0 (0.0–3.0)
Chills	0.0 (0.0–1.0)	1.5 (0.0–5.0)	0.0 (0.0–1.0)
Nausea	0.0 (0.0–0.0)	0.0 (0.0–0.0)	0.0 (0.0–0.0)
Diarrhea	0.0 (0.0–0.0)	0.0 (0.0–0.3)	0.0 (0.0–0.0)
Muscle pain	1.0 (0.0–3.0)	1.0 (0.0–2.0)	1.0 (0.0–2.0)
Joint pain	0.0 (0.0–2.0)	0.5 (0.0–4.0)	0.0 (0.0–2.0)
Sleep duration from actigraphy			
3 days before the booster vaccination	368.3 (300.0–428.0)	345.7 (315.7–383.7)	363.0 (308.2–409.2)
3 days after the booster vaccination	398.7 (315.5–457.3)	434.3 (364.8–532.7)	411.0 (357.7–525.8)
7 days after the booster vaccination	380.9 (305.9–417.8)	410.6 (371.9–417.5)	391.9 (339.9–417.3)
3 days before the first vaccination	338.7 (269.5–416.7)	379.0 (345.8–403.3)	376.3 (296.0–408.2)
3 days after the first vaccination	386.0 (341.2–456.3)	412.3 (389.2–455.3)	398.0 (360.3–455.7)
Whole observational period^b^	372.4 (315.2–409.3)	384.4 (357.0–405.7)	380.1 (332.8–404.9)
Sleep duration from sleep diaries			
3 days before the booster vaccination	422.5 (380.0–461.3)	412.5 (353.8–458.8)	415.0 (380.0–460.0)
3 days after the booster vaccination	450.0 (407.5–523.8)	510.0 (472.5–580.0)	470.0 (416.3–538.8)
7 days after the booster vaccination	431.8 (410.4–469.8)	456.4 (429.1–492.9)	437.1 (413.0–487.0)
3 days before the first vaccination	411.2 (355.0–456.2)	437.5 (360.0–477.5)	416.3 (360.0–460.0)
3 days after the first vaccination	440.0 (417.5–475.0)	480.0 (420.0–575.0)	442.5 (420.0–493.8)
Whole observational period^b^	423.9 (406.8–448.1)	440.4 (399.0–475.7)	426.7 (405.7–463.2)
Time of day of the first vaccination	14: 30 (11:11–15:48)	13:52 (12:26–14:30)	14:00 (11:18–14:30)
Time of day of the booster vaccination	14:15 (10:50–15:52)	13:52 (12:22–14:30)	13:52 (11:03–15:30)
Antibody titer against COVID-19 S protein	2230.0 (1177.5–3407.5)	3980.0 (2865.0–7332.5)	2650.0 (1632.5–3930.0)
Log-transformed antibody titer	3.35 (3.07–3.53)	3.60 (3.46–3.84)	3.42 (3.21–3.59)
Log- and Z-transformed antibody titer	0.18 (−0.69–0.75)	−0.25 (−0.77–0.65)	0.05 (−0.72–0.69)

Values reported are frequencies (percentages) or medians (interquartile ranges).

^a^Sum of the grades of reactogenicity, ranging from 0 to 3, including fever, dizziness, fatigue, headache, chills, nausea, diarrhea, muscle pain, and joint pain for 8 days after vaccination.

^b^Period from the date of consent to the date of blood sampling. The date of consent ranged from 1 day to 25 days before the first vaccination, and the blood sampling date ranged from 7 to 23 days after the booster vaccination.

COVID-19, coronavirus disease 2019.

Univariable linear regression analysis revealed that actigraphy-measured objective sleep durations 3 and 7 days after the booster vaccination were significantly correlated with the antibody titer. However, objective sleep duration 3 days before the booster vaccination, 3 days before the first vaccination, 3 days after the first vaccination, or over the whole observational period was not significantly correlated with the antibody titer. Moreover, none of the sleep-diary-measured subjective sleep duration values was significantly correlated with the antibody titer (**Table 2**).

**Table 2:** Univariate linear regression analysis of log- and Z-transformed antibody titers and sleep measures and covariates.

Variables	Unstandardized coefficient		Standardized coefficient	p
	B (95% CI)	SE	Beta	
Type of vaccine	0.000 (−0.639–0.639)	0.318	0.000	>0.99
Sex	0.207 (−0.390–0.804)	0.297	0.103	0.49
Age	0.017 (−0.018–0.051)	0.017	0.142	0.34
Systemic reactogenicity	0.027 (0.002–0.053)	0.013	0.300	0.04
Time of day of the first vaccination	0.000 (0.000–0.000)	0.000	–0.103	0.49
Time of day of the booster vaccination	0.000 (0.000–0.000)	0.000	–0.093	0.53
Average sleep duration from actigraphy				
3 days before the booster vaccination	0.000 (−0.003–0.003)	0.001	0.030	0.85
3 days after the booster vaccination	0.003 (0.001–0.005)	0.001	0.364	0.01
7 days after the booster vaccination	0.003 (0.000–0.007)	0.002	0.310	0.04
3 days before the first vaccination	0.001 (−0.002–0.005)	0.002	0.102	0.51
3 days after the first vaccination	0.001 (−0.002–0.004)	0.002	0.105	0.48
Whole observational period	0.001 (−0.003–0.006)	0.002	0.086	0.56
Average sleep duration from sleep diaries				
3 days before the booster vaccination	−0.112 (−0.330–0.105)	0.108	−0.152	0.30
3 days after the booster vaccination	0.016 (−0.144–0.176)	0.079	0.030	0.84
7 days after the booster vaccination	−0.058 (−0.330–0.214)	0.135	−0.024	0.67
3 days before the first vaccination	−0.028 (−0.142–0.087)	0.057	−0.071	0.63
3 days after the first vaccination	0.075 (−0.126–0.275)	0.100	0.110	0.46
Whole observational period	−0.185 (−0.497–0.128)	0.155	−0.172	0.24

B, the unstandardized Coefficients. CI, confidence interval. SE, the standard error. Beta, the standardized Coefficients.

Multivariable linear regression analysis revealed that the objective average sleep duration 3 days after the booster vaccination was significantly and independently correlated with the antibody titer (B=0.003; 95% confidence interval, 0.000–0.005; Beta=0.337; p=0.02) after controlling for age, sex, type of vaccine, and total score for systemic reactogenicity (**Table 3**). In this multivariable model, although the total score for systemic reactogenicity was significantly correlated with the antibody titer in the univariable analysis, it was not significant in the multivariable analysis. The exploratory analysis showed that objective average sleep duration 7 days after the booster vaccination was also significantly correlated with the antibody titer, even after controlling for covariates.

**Table 3:** Multivariable linear regression analysis of associations between mRNA vaccine-induced antibody titers and average sleep duration.

Measure	Model	Crude				Model 1					Model 2				
	Days relative to the booster vaccination	B (95% CI)	SE	Beta	p	B (95% CI)	SE	Beta	R^2^	p	B (95% CI)	SE	Beta	R^2^	p
Actigraphy	3 days before	2.8×10^−4^ (−0.003–0.003)	0.001	0.030	0.85										
	3 days after	0.003 (0.001–0.005)	0.001	0.364	0.01	0.003 (0.001–0.005)	0.001	0.448	0.210	0.004	0.003 (4.7×10^−4^–0.005)	0.001	0.377	0.270	0.02
	7 days after	0.003 (2.3×10^−4^–0.007)	0.002	0.310	0.04	0.004 (0.001–0.008)	0.002	0.395	0.171	0.01	0.004 (0.001–0.007)	0.002	0.381	0.284	0.01
	Whole observational period	0.001 (−0.003–0.006)	0.002	0.086	0.56										
Sleep diary	3 days before	−0.112 (−0.330–0.105)	0.108	−0.152	0.30										
	3 days after	0.016 (−0.144–0.176)	0.079	0.030	0.84										
	7 days after	−0.058 (−0.330–0.214)	0.135	−0.024	0.67										
	Whole observational period	−0.185 (−0.497–0.128)	0.155	−0.172	0.24										

Model 1: adjusted for age and sex.

Model 2: adjusted for model 1 + the type of vaccine and total score for systemic reactogenicity.

B, the unstandardized Coefficients. CI, confidence interval. SE, the standard error. Beta, the standardized Coefficients.

## 4 Discussion

This study demonstrated a correlation between longer objective sleep duration following booster vaccination and higher antibody titers acquired after SARS-CoV-2 mRNA vaccination in healthy adults. In addition to sleep duration 3 days post-vaccination, 7 days of adequate objective sleep following booster vaccination was also considered beneficial for antibody expression. Studies with inactivated vaccines have shown the importance of sleep before and after vaccination (11). In contrast, our study underscores the significance of securing adequate sleep for an extended period following mRNA vaccination to achieve higher antibody titers. This may be due to the nature of mRNA vaccines, which expose the host to antigens for a longer period of time (6).

The vaccine-induced antibody titer correlated with actigraphy-recorded objective sleep duration but not with subjective sleep duration measured using a sleep diary. These results agree with the findings of a previous study showing that subjective sleep duration does not affect the antibody titer acquired following mRNA vaccination (12). The results of this study are also consistent with those of Prather et al.’s study that used anti-hepatitis B vaccine (5) and of a recent meta-analysis (11), suggesting that measuring objective (but not subjective) sleep duration is essential when evaluating antibody acquisition. The sensitivity of subjective sleep to sleep duration (30), mood (31), and night-to-night variability (32) may attenuate the effect of sleep duration on immune response. Unlike previous interventions (2, 3), this study was observational, in which the participants were allowed to sleep freely. Future trials are warranted to determine whether more restrictive sleep deprivation negatively affects the acquisition of antibody titers to mRNA vaccines.

In this study, the correlation between systemic reactogenicity and the antibody titer was insignificant after controlling for covariates, including sleep duration. These results imply that a higher level of reactogenicity could result from elevated antibody titers through prolonged sleep. Although previous studies have suggested a correlation between severe reactogenicity and higher antibody acquisition (22–24), these studies did not examine the influence of sleep habits after vaccination against SARS-CoV-2. One study reported prolonged sleep for at least 5 days after mRNA vaccination, excluding the day of vaccination (33). Therefore, further interventional studies are needed to elucidate the causal relationship among antibody acquisition, reactogenicity, and sleep duration.

In this study, no significant relationship between antibody titer and time of day of vaccination was found. Although previous studies with inactivated vaccines have suggested a potential relationship between the time of day of vaccination and antibody acquisition (25), the results of observational studies using the mRNA vaccine have been inconsistent. One study reported that afternoon vaccination resulted in higher antibody titers (34), while another study suggested that the timing of vaccination had no effect on antibody titers (35). The cause of this disparity is not clear, but the nature of long-acting feature of the mRNA vaccine may have mitigated the effect of the time of day of vaccination (6). As stated in a review, in 85% of studies, drug half-lives of less than 15 hours showed dose-time dependence compared with 39% of studies that used longer-acting drugs (36). Moreover, the effect of time of day of vaccination in this study may have been too subtle to be detected given the statistical power of our study. Further research is warranted to elucidate the relationship between the timing of vaccination and the immune response induced by the mRNA vaccine.

The present study showed that sleep duration around the first vaccination did not correlate with antibody titers at two weeks after the second vaccination. This finding contradicts a previous study using inactivated vaccine, which showed that sleep around the first vaccination have affected longer-term vaccine response (5). The reason for the lack of association between sleep duration after the first vaccination and antibody titers remains unclear; however, it is possible that differences in immune response between inactivated and mRNA vaccines may have influenced for the discrepancy between inactivated and mRNA vaccines in response to sleep duration around the first vaccination. For example, the declining nature of antibody titers acquired with the SARS-CoV-2 vaccine over time (37) may have influenced this discrepancy. Although there may be an association exclusively between recent vaccine administration and sleep duration, the lack of antibody titer measurements after the first dose of vaccine prevents a clear determination of causality. Further studies with more frequent, longer-term monitoring of vaccine response are needed to clarify this discrepancy.

This study has some limitations. Government and hospital policies banning long-distance travel due to the COVID-19 pandemic prevented us from recruiting a sufficient number of participants. The limited pool of potential participants may have affected the power of our analysis and restricted our ability to focus on each of the mRNA vaccines when testing our hypothesis. Next, actigraphy data loss exceeded 20% during two distinct periods: 2 or more days before the first vaccination and 12 days after the booster. Careful consideration should be given to the interpretation of data during these periods. Third, the present study did not include older people, who are believed to be more susceptible to infections. In general, older individuals sleep less and have a weaker response to vaccination (18, 38). Further studies are needed to elucidate the relationship between age, sleep duration, and vaccine response.

In conclusion, longer objective sleep duration after booster vaccination may maximize the benefit of mRNA vaccines against SARS-CoV-2. Follow-up studies with larger samples are warranted, as this is a preliminary study. The mRNA vaccines can potentially be applied against COVID-19 and various other diseases, such as HIV/AIDS and cancer (39). The impact of adequate sleep duration after mRNA vaccination on antibody titer acquisition should be verified in a randomized controlled trial.

## Data availability statement

The datasets presented in this article are not readily available because Data available upon request due to ethical reasons. The data underlying this study cannot be published or shared externally without permission granted by the Ethics Committee of the National Center of Neurology and Psychiatry. Please contact the corresponding authors for questions. Requests to access the datasets should be directed to Kentaro Matsui, matsui.kentaro@ncnp.go.jp.

## Ethics statement

The studies involving humans were approved by The Ethics Committee of the National Centre for Neurology and Psychiatry Hospital. The studies were conducted in accordance with the local legislation and institutional requirements. The participants provided their written informed consent to participate in this study.

## Author contributions

MI and KM had full access to all of the data in the study and take responsibility for the integrity of the data and the accuracy of the data analysis. Concept and design: MI, KM, and KK. Acquisition, analysis, or interpretation of data: All authors. Drafting of the manuscript: MI and KM. Critical revision of the manuscript for important intellectual content: All authors. Statistical analysis: MI and KM. Obtained funding: MI and KK. Administrative, technical, or material support: SK and KK. Supervision: KK. All authors contributed to the article and approved the submitted version.
