# Supplementary material for: Association between sleep duration and antibody acquisition after mRNA vaccination against SARS-CoV-2
Source: Front Immunol. 2023 Dec 11;14:1242302. doi: 10.3389/fimmu.2023.1242302 (PMC10750410; doi:10.3389/fimmu.2023.1242302)
Supplement: Supplementary file 1 [file DataSheet_1.docx]

Supplementary Material

Association between sleep duration and antibody acquisition after mRNA vaccination against SARS-CoV-2

Muneto Izuhara, Kentaro Matsui^*^, Takuya Yoshiike, Aoi Kawamura, Tomohiro Utsumi, Kentaro Nagao, Ayumi Tsuru, Rei Otsuki, Shingo Kitamura, Kenichi Kuriyama^*^

*** Correspondence:** Kenichi Kuriyama: kenichik@ncnp.go.jp; Kentaro Matsui: matsui.kentaro@ncnp.go.jp

**
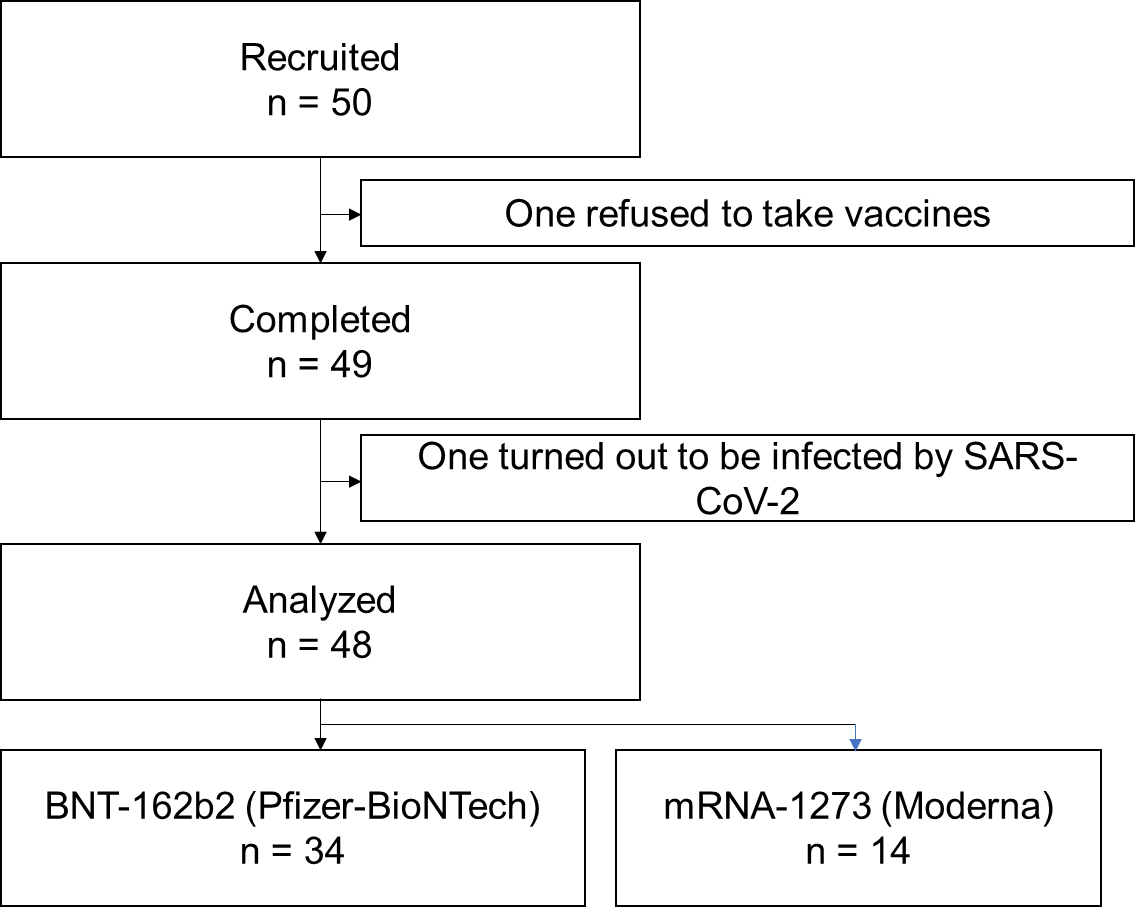
**

**Supplementary Figure 1.** Flow diagram of participant enrollment. SARS-CoV-2, severe acute respiratory syndrome coronavirus 2.


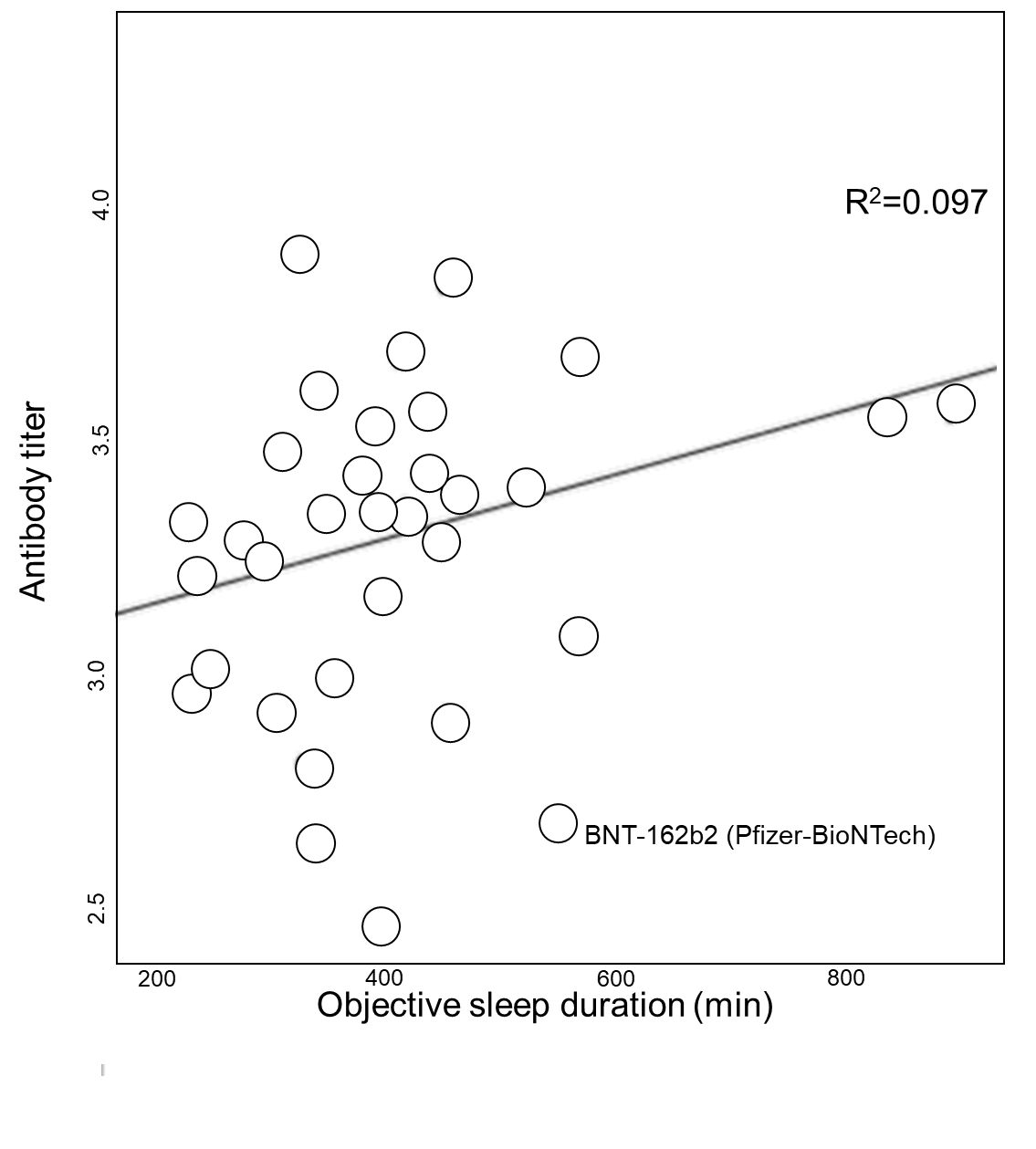


**Supplementary Figure 2.** Average sleep duration 3 days following booster vaccination and log-transformed antibody titers for BNT-162B2 (Pfizer-BioNTech).


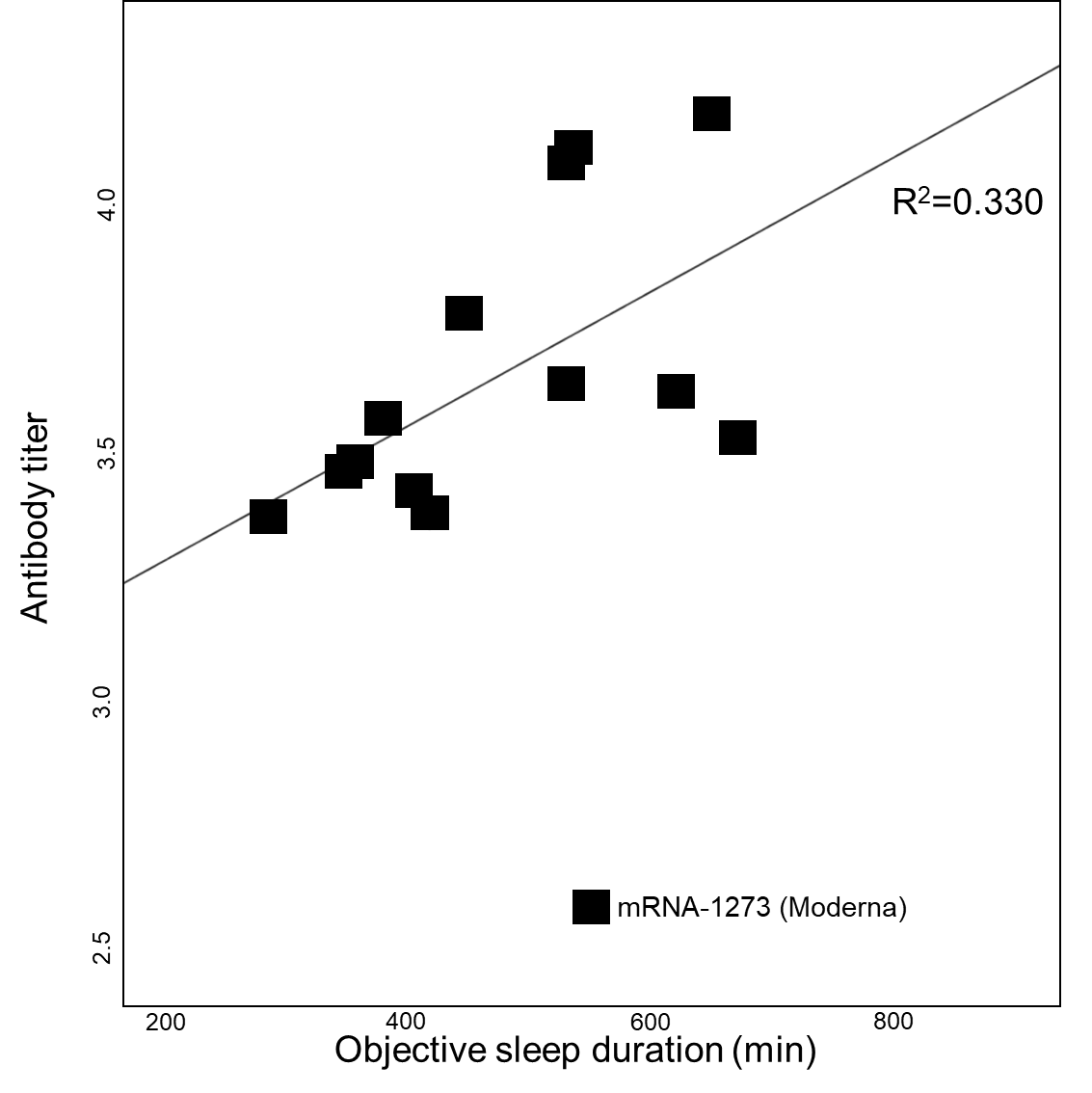


**Supplementary Figure 3.** Average sleep duration 3 days following booster vaccination and log-transformed antibody titers for mRNA-1273 (Moderna).

**Supplementary Table 1.** Missing actigraphy data for each day

| The rate of participants without actigraphy data for each day [%] | Days before/after the vaccination | | | | | | | | | | | | | | |
| --- | --- | --- | --- | --- | --- | --- | --- | --- | --- | --- | --- | --- | --- | --- | --- |
|  | 0 | 1 | 2 | 3 | 4 | 5 | 6 | 7 | 8 | 9 | 10 | 11 | 12 | 13 | 14 |
| Before the first vaccination |  | 8.3 | 31.3 | 45.8 | 54.2 | 64.6 | 70.8 | 72.9 | 79.2 | 79.2 | 87.5 | 87.5 | 91.7 | 91.7 | 93.8 |
| After the first vaccination | 4.2 | 6.3 | 10.4 | 8.3 | 8.3 | 8.3 | 12.5 | 8.3 | 14.6 | 12.5 | 18.8 | 12.5 | 16.7 | 8.3 | 14.6 |
| Before the booster vaccination |  | 16.7 | 14.6 | 14.6 | 16.7 | 12.5 | 10.4 | 14.6 | 8.3 | 18.8 | 12.5 | 16.7 | 12.5 | 12.5 | 6.3 |
| After the booster vaccination | 6.3 | 6.3 | 12.5 | 14.6 | 14.6 | 6.3 | 10.4 | 16.7 | 14.6 | 16.7 | 14.6 | 18.8 | 31.3 | 43.8 | 64.6 |

**STROBE Statement—Checklist of items that should be included in reports of cohort studies**

|  | Item No | Recommendation | Authors’ comment |
| --- | --- | --- | --- |
| Title and abstract | 1 | (*a*) Indicate the study’s design with a commonly used term in the title or the abstract | The study design is described in the abstract. |
|  |  | (*b*) Provide in the abstract an informative and balanced summary of what was done and what was found | The abstract contains what was done and what was found. |
| Introduction | | |  |
| Background/rationale | 2 | Explain the scientific background and rationale for the investigation being reported | The scientific background and rationale for the investigation are described in the Introduction. |
| Objectives | 3 | State-specific objectives, including any prespecified hypotheses | The objective is stated at the end of the Introduction. |
| Methods | | |  |
| Study design | 4 | Present key elements of study design early in the paper | The study design is described at the beginning of the Materials and methods. |
| Setting | 5 | Describe the setting, locations, and relevant dates, including periods of recruitment, exposure, follow-up, and data collection | The settings are in the Materials and methods. |
| Participants | 6 | (*a*) Give the eligibility criteria and the sources and methods of selection of participants. Describe methods of follow-up | The eligibility criteria and selection of participants are described in the Participants and Supplementary Figure 1. |
|  |  | (*b*) For matched studies, give matching criteria and the number of exposed and unexposed | Not relevant. |
| Variables | 7 | Clearly define all outcomes, exposures, predictors, potential confounders, and effect modifiers. Give diagnostic criteria, if applicable | The main outcome (relationship between the antibody titer and sleep duration) and covariates are described in the Materials and methods section. |
| Data sources/ measurement | 8* | For each variable of interest, give sources of data and details of methods of assessment (measurement). Describe comparability of assessment methods if there is more than one group | Sleep measurements and data analysis are described in the Materials and methods section. |
| Bias | 9 | Describe any efforts to address potential sources of bias | As stated in the Materials and methods section, variables related to antibody titers were collected and analyzed as covariates. |
| Study size | 10 | Explain how the study size was arrived at | The sample size calculation is described in the Sample size calculation. |
| Quantitative variables | 11 | Explain how quantitative variables were handled in the analyses. If applicable, describe which groupings were chosen and why | Average sleep durations were calculated for 7 days after each vaccination for exploratory analysis and the main analysis of 3 days. |
| Statistical methods | 12 | (*a*) Describe all statistical methods, including those used to control for confounding | Statistical methods are described in the Data analysis. |
|  |  | (*b*) Describe any methods used to examine subgroups and interactions | No subgroup analysis was conducted. |
|  |  | (*c*) Explain how missing data were addressed | Mean substitution was performed. |
|  |  | (*d*) If applicable, explain how the loss to follow-up was addressed | Reasons for exclusion are described in the Materials and methods section. |
|  |  | (*e*) Describe any sensitivity analyses | As stated in the Data analysis, sensitivity analysis was conducted for longer days of average sleep duration. |
| Results | | |  |
| Participants | 13* | (a) Report numbers of individuals at each stage of study—eg numbers potentially eligible, examined for eligibility, confirmed eligible, included in the study, completing follow-up, and analyzed | The number of participants at each study stage is described at Participant and Supplementary Figure 1. |
|  |  | (b) Give reasons for non-participation at each stage | Not relevant. |
|  |  | (c) Consider the use of a flow diagram | The flow diagram is shown in Supplementary Figure 1. |
| Descriptive data | 14* | (a) Give characteristics of study participants (eg demographic, clinical, social) and information on exposures and potential confounders | Participant characteristics are described in Table 1. |
|  |  | (b) Indicate the number of participants with missing data for each variable of interest | Missing actigraphy-measured sleep data are described in Supplementary Table 1. |
|  |  | (c) Summarize follow-up time (eg, average and total amount) | The entire observational period is described in Table 1. |
| Outcome data | 15* | Report numbers of outcome events or summary measures over time | Antibody titers and sleep duration are described in Table 1, as well as in the Results. |
| Main results | 16 | (*a*) Give unadjusted estimates and, if applicable, confounder-adjusted estimates and their precision (e.g., 95% confidence interval). Make clear which confounders were adjusted for and why they were included | Unadjusted and confounder-adjusted estimates are described in Table 3. |
|  |  | (*b*) Report category boundaries when continuous variables were categorized | Average sleep duration of 3 days before and after the vaccination and 7 days after the vaccination was examined.  The main outcome was evaluated over 3 days. Sensitivity analyses were conducted for a 7-day period (Table 3). |
|  |  | (*c*) If relevant, consider translating estimates of relative risk into absolute risk for a meaningful time period | No relative risk was calculated. |
| Other analyses | 17 | Report other analyses done—e.g. analyses of subgroups and interactions and sensitivity analyses | The results of the sensitivity analysis (7-day sleep duration analysis) are described in Table 3. |
| Discussion | | |  |
| Key results | 18 | Summarize key results with reference to study objectives | Key results are summarized at the beginning of the Discussion. |
| Limitations | 19 | Discuss the limitations of the study, taking into account sources of potential bias or imprecision. Discuss both the direction and magnitude of any potential bias | The limitations are described at the end of the Discussion. |
| Interpretation | 20 | Give a cautious overall interpretation of results considering objectives, limitations, the multiplicity of analyses, results from similar studies, and other relevant evidence | The overall interpretation is stated in the end of the Discussion. |
| Generalizability | 21 | Discuss the generalizability (external validity) of the study results | Generalizability is discussed in the Discussion. |
| Other information | | |  |
| Funding | 22 | Give the source of funding and the role of the funders for the present study and, if applicable, for the original study on which the present article is based | The funding source is listed in the Funding. |

*Give information separately for exposed and unexposed groups.

Note: An Explanation and Elaboration article discusses each checklist item and gives methodological background and published examples of transparent reporting. The STROBE checklist is best used in conjunction with this article (freely available on the Web sites of PLoS Medicine at http://www.plosmedicine.org/, Annals of Internal Medicine at http://www.annals.org/, and Epidemiology at http://www.epidem.com/). Information on the STROBE Initiative is available at http://www.strobe-statement.org.
